# Potential clinical utility of liquid biopsies in ovarian cancer

**DOI:** 10.1186/s12943-022-01588-8

**Published:** 2022-05-11

**Authors:** Jie Wei Zhu, Parsa Charkhchi, Mohammad R. Akbari

**Affiliations:** 1 Women’s College Research Institute, Women’s College Hospital, University of Toronto, 76 Grenville St, Toronto, ON M5S 1B2 Canada; 2 Department of Medicine, McMaster University, Hamilton, ON Canada; 3 Institute of Medical Science, Faculty of Medicine, University of Toronto, Toronto, ON Canada; 4 Dalla Lana School of Public Health, University of Toronto, Toronto, ON Canada

## Abstract

Ovarian cancer (OC) is the most lethal gynecologic malignancy worldwide. One of the main challenges in the management of OC is the late clinical presentation of disease that results in poor survival. Conventional tissue biopsy methods and serological biomarkers such as CA-125 have limited clinical applications. Liquid biopsy is a novel sampling method that analyzes distinctive tumour components released into the peripheral circulation, including circulating tumour DNA (ctDNA), circulating tumour cells (CTCs), cell-free RNA (cfRNA), tumour-educated platelets (TEPs) and exosomes. Increasing evidence suggests that liquid biopsy could enhance the clinical management of OC by improving early diagnosis, predicting prognosis, detecting recurrence, and monitoring response to treatment. Capturing the unique tumour genetic landscape can also guide treatment decisions and the selection of appropriate targeted therapies. Key advantages of liquid biopsy include its non-invasive nature and feasibility, which allow for serial sampling and longitudinal monitoring of dynamic tumour changes over time. In this review, we outline the evidence for the clinical utility of each liquid biopsy component and review the advantages and current limitations of applying liquid biopsy in managing ovarian cancer. We also highlight future directions considering the current challenges and explore areas where more studies are warranted to elucidate its emerging clinical potential.

## Introduction

Ovarian cancer is the third most common gynecologic malignancy worldwide and is associated with the highest mortality rates among gynecologic cancers [1]. Each year, more than 240,000 new cases are diagnosed, and 150,000 women die from ovarian cancer, with five-year survival rates below 45% [2]. Ovarian cancer encompasses a heterogeneous group of neoplasms classified based on distinctive histopathological and molecular characteristics. Epithelial ovarian cancer (EOC) is the most common type of ovarian cancer that is further classified into four major subtypes based on tumour cell morphology: serous, endometrioid, clear cell, and mucinous [3].

**Fig. 1:**
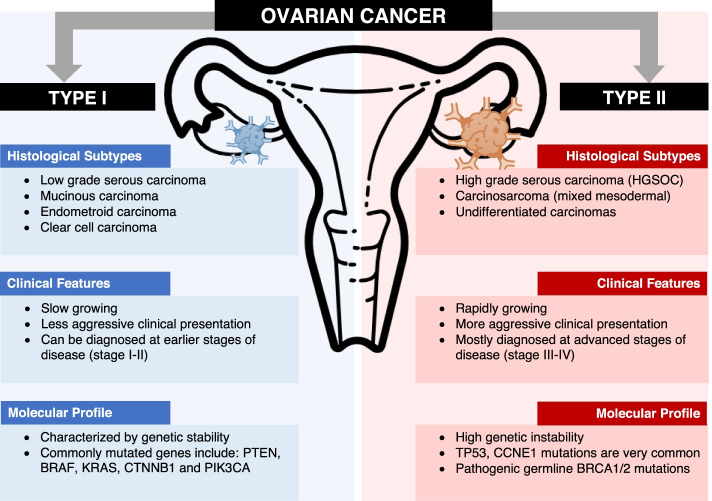
Schematic representation of ovarian cancer classification into Type I and Type II tumours based on histology, clinical features, and molecular profile with commonly associated mutations. Type I tumours tend to be slow growing, less aggressive, and more likely to be diagnosed at earlier stages of disease associated with genetic stability. Type II tumours usually present with more aggressive, rapid growing disease that is diagnosed in more advanced stages, and are associated with a higher degree of genetic instability

Ovarian cancer has also been classified into two subtypes with distinct molecular profiles and clinical courses (Fig. 1). Type I tumours are low-grade, more indolent, and less aggressive tumours that are characterized by mutations in mitogen-activated protein kinase (MAPK) regulator pathways (e.g. KRAS or BRAF) [4]. In contrast, Type II tumours such as high-grade serous ovarian cancer (HGSOC) are aggressive and have high genetic instability. These are associated with high mutation rates in *TP53*, somatic and germline *BRCA1/2 *and other homologous recombination genes [5]. Identifying the unique tumour mutational profile can guide treatment decisions and the selection of appropriate targeted therapy. For example, polyadenosine diphosphate (ADP)-ribose polymerase inhibitor (PARPi) treatment confers a significant progression-free survival (PFS) benefit in patients with a germline or somatic *BRCA1/2 *mutation by causing an accumulation of double-stranded DNA breaks and cell death [6–8].

The high morbidity and mortality in ovarian cancer are related to the late diagnosis of disease and decreased effectiveness of surgical or pharmacological therapies. Due to the late onset of symptoms and their nonspecific nature, up to 75% of ovarian cancer cases are diagnosed with advanced disease, of which only about 20% will live up to 5 years from the time of diagnosis [9, 10]. The current standard of care for ovarian cancer is cytoreductive surgery followed by platinum-based chemotherapy [11]. Although patients initially respond well to treatment, most patients with advanced ovarian cancer relapse and develop chemoresistance within a few years [12].

There has been an ongoing search for diagnostic, prognostic, or predictive biomarkers to improve ovarian cancer management in the last few decades. Although CA-125 is currently the best-characterized biomarker in ovarian cancer, its sensitivity, specificity, and survival benefit are insufficient for routine screening purposes [12, 13]. Temporal monitoring of CA-125 during follow-up has also not demonstrated benefit in overall survival (OS) [12]. Similarly, tissue biopsies are not feasible as they are highly invasive and only provide localized sampling with limited sensitivity. These shortcomings of existing screening and detection methods have resulted in a continued search for more specific and sensitive biomarkers for ovarian cancer.

**Fig. 2:**
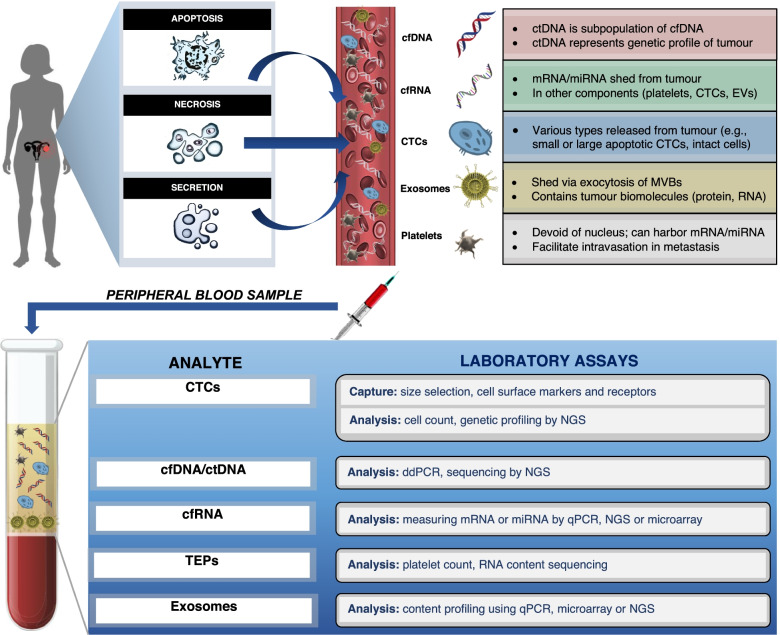
Overview of the liquid biopsy process, from hypothesized mechanisms of tumour release of liquid biopsy components, to laboratory analysis techniques. Tumour biomarkers are first released and enter the circulation via one of three main mechanisms: apoptosis, necrosis, or secretion. Liquid biopsy involves the collection and analysis of five distinctive tumour components from peripheral blood samples: cell-free nucleic acids (cfDNA/ctDNA, cfRNA), CTCs, exosomes and tumour educated platelets. Tumour components in peripheral blood samples are then captured and analyzed using their corresponding laboratory assays. cfDNA: circulating free DNA, ctDNA: circulating tumour DNA, cfRNA: cell-free RNA, CTCs: circulating tumour cells, TEPs: tumour educated platelets, NGS: next generation sequencing, qPCR: quantitative polymerase chain reaction

In the last decade, liquid biopsies that measure various tumour components, including circulating tumour DNA (ctDNA), cell-free RNA (cfRNA), circulating tumour cells (CTCs), tumour educated platelets (TEPs) and exosomes, have become recognized as a method for molecular screening and earlier diagnosis of ovarian cancer (Fig. 2). Compared to traditional tissue biopsies, liquid biopsy is minimally invasive and serial blood samples can be collected over time to monitor cancer progression in real-time. This review discusses the advantages and current limitations of liquid biopsy in the management of ovarian cancer. It will also explore different components and techniques of liquid biopsy, and its utility in ovarian cancer diagnosis, prognosis, and clinical monitoring of treatment response or recurrence.

## Liquid biopsy components

### CTC: physiologic characteristics and analysis

CTCs are cancer cells found in peripheral blood that intravasate or are passively shed from a primary or metastatic solid tumour site. Several analytic methods for CTC isolation have been developed and validated for ovarian cancer that are based on various biological (i.e., positive epithelial markers, negative hematopoietic markers) or physical properties (i.e., size, density, deformability, electric charges, and invasive capacity) [14–29]. The ability to detect CTCs in the bloodstream has important prognostic implications in ovarian cancer for identifying potential micrometastasis, pre-neoplastic lesions, tumour heterogeneity, and tumour evolution over time [30–33].

Isolation of CTCs from peripheral blood samples is technically challenging given the low concentration with approximately 1 CTC in 1,000,000 circulating cells [34–36]. Following release from the primary tumour, CTCs overcome several obstacles to survive in the systemic vasculature and spread to distant organs [37]. First, tumour cells shed from solid tumours often traverse the endothelium to enter the circulation by undergoing the epithelial-to-mesenchymal transition (EMT) process. EMT is a phenotypic transformation of epithelial cells with loss of polarity, morphology, and cell markers such as the epithelial cell adhesion molecule (EpCAM), to gain the migratory and invasive properties of mesenchymal cells [38]. After dissociation from a primary model V_2 _carcinoma (established from skin carcinoma of cottontail rabbits) site of 1 cm, approximately one million CTCs intravasate via dermal invasion into the peripheral circulation each day, of which < 1% typically remain viable for metastasis [39]. Most CTCs undergo apoptosis or necrosis due to the profound environmental challenges in the bloodstream such as starvation, shear stress, and immunological detection [40]. Only a small proportion of CTCs can survive through upregulation of several signalling pathways, including increased secretion of growth factors, downregulation of death receptors, and over-expression of anti-apoptotic ligands [39]. CTCs must also evade natural immune system defences and natural killer (NK) cell recognition. According to the adaptive immune resistance theory, tumour cells avoid activation of the antitumor response from NK cells and T cells by upregulating the naturally occurring programmed death-ligand 1 (PD-L1). They also avoid phagocytosis by macrophages through the upregulation of CD47 [41–43].

Currently, the CellSearch detection system is the most widely used isolation strategy with US Food and Drug Administration (FDA) approval. CellSearch uses an immunoaffinity-based isolation strategy to identify CTCs based on positive EpCAM expression [44]. However, the application of CellSearch in ovarian cancer may be limited in the setting of low EpCAM expression. In a study with newly diagnosed or recurrent ovarian cancer patients, Liu et al. found no correlation between serial CTC enumeration by the CellSearch system and clinical outcomes [15]. One possible explanation is the downregulation of EpCAM during EMT or the heterogeneous expression of cell surface markers in ovarian cancer [15]. Obermayr et al. also reported EpCAM expression in a small proportion of EOC patients [21]. The researchers used RT-qPCR to analyze EpCAM expression of CTCs isolated using density gradient centrifugation from 216 EOC patients before and after primary treatment compared to 39 healthy controls. The researchers reported EpCAM expression in only 8% of patients at baseline before treatment and 4% for patients after six months of adjuvant chemotherapy.

To overcome these limitations, alternative approaches targeting various biophysical properties of CTCs such as cell size and invasive potential have been developed [14, 29]. For example, Yang et al. recently used a technique called CanPatrol enrichment, in which CTCs were filtered through 8-μm porous membranes and detected using RNA in situ hybridization (RNA-ISH). CTC subpopulations were identified using epithelial (EpCAM and CK8/18/19), mesenchymal (vimentin and Twist), and epithelial-mesenchymal hybrid markers [14]. The researchers used hybrid markers and found a mean CTC count of 8.70 ± 5.69 detected in 5 mL of blood among stage 1-IV EOC patients that was significantly higher compared to controls with benign gynecologic diseases (1.04 ± 0.73). Multivariate analyses demonstrated both higher CTC counts and higher percentage of mesenchymal CTC were independent prognostic factors for significantly lower OS (*p* = 0.012 and *p* = 0.009 respectively). Fan et al. proposed another novel enrichment method utilizing the unique property that blood containing CTCs will invade and ingest Cell Adhesion Matrix (CAM) while non-tumour and dead tumour cells do not [29]. The researchers used a cell invasion assay that enriches and identifies tumour cells based on CAM invasion (CAM +) and expression of standard epithelial markers (Epi +) to analyze peripheral blood samples of 71 suspected ovarian cancer patients. The study found a significantly higher mean CTC count in stage III/IV patients at 41.3 CTCs/ml compared to 6.0 CTCs/ml in stage I/II patients and 0 CTCs/ml in benign patients (*p*-value = 0.001). Kaplan Meier analysis showed a significantly lower disease-free survival in patients with detectable CTCs with a median survival of 15.0 months compared to 35.0 months in those without detected CTCs (*p*= 0.042). Other novel techniques include modified immunoaffinity-based strategies targeting several ligands at once (e.g. EpCAM, folate receptor alpha, Human epidermal growth factor receptor 2) and nanoparticles conjugated with the antibody against Mucin 1 (MUC1) [45, 46]. Given the rarity of CTCs in peripheral circulation despite their prognostication potential, further studies are required to optimize the detection and isolation of CTCs in ovarian cancer.

### cfDNA/ctDNA: physiological characteristics and analysis

Normally, plasma contains cfDNA that is passively released from necrotic or apoptotic cells, while ctDNA is the cfDNA secreted from cancer cells. In healthy individuals, cfDNA concentrations are elevated following tissue damage such as intense exercise, inflammation, sepsis, surgery, radiotherapy, trauma, or during pregnancy [47–49].

Compared to CTCs, cfDNA concentrations are higher in blood, making them suitable targets for liquid biopsy [50]. Tumours harbour unique somatic genetic alterations that help in distinguishing ctDNA from noncancerous cfDNA [47, 48]. The majority of cfDNA is expected to originate from healthy cells, while a variable amount of cfDNA (0.01–93%, depending on the tumour size) can originate from cancer cells (ctDNA) [51, 52]. However, a popular hypothesis posits that a large fraction of cfDNA is released from cells in the tumour microenvironment that were destroyed due to hypoxia or the antitumour response [53]. Recent studies confirmed that cfDNA levels in the blood are higher among ovarian cancer patients with an average of 180 ng/mL compared to 30 ng/mL in healthy controls or individuals with benign ovarian pathologies [54–56]. Therefore, increased amounts of cfDNA may serve as a diagnostic tool for ovarian cancer, while genomic analysis of ctDNA may provide valuable prognostic and predictive information [57, 58].

The mechanism of cfDNA released from cells into the circulation remains unclear, although apoptosis and necrosis are the most widely accepted hypotheses based on cfDNA properties. Previous studies have estimated the size of cfDNA to vary from ~ 40–200 base pairs (bp), with a peak at around 166 bp [53, 59–61]. Agarose gel electrophoresis to separate extracted cfDNA has found fragment ladders ranging from 160 bp up to 21 kbp [61, 62]. The size of these fragments corresponds primarily to mono- and oligonucleosomes that are characteristic of caspase-dependent cleavage during apoptosis [59, 61–63]. In contrast, DNA fragments larger than 10 kb are thought to be a result of necrotic cell death in tumours with different kinetics and amount of cfDNA released from different necrosis-inducing agents [64–66]. However, this theory has been called into question following studies reporting that radiation therapy, which typically induces tissue necrosis, results in a reduction of cfDNA levels by up to 90% in the plasma of cancer patients [67, 68]. Other proposed cfDNA release mechanisms include active secretion in living cells with the expulsion of nuclei, phagocytosis, neutrophil extracellular trap release (NETosis), and excision repair [69–74].

Once released into the bloodstream, the size, integrity, and half-life of cfDNA have important clinical implications in diagnosis and tumour detection. One challenge currently is the small amount of ctDNA in the blood compared to cfDNA released from normal cells, particularly when the tumour size is small. Since a significant proportion of ctDNA is released from necrotic cancer cells, the cfDNA size in cancer patients is generally longer than those of healthy individuals. However, the length of ctDNA released from apoptotic cancer cells is shorter than cfDNA released from the normal cells due to apoptosis, with a mean of 133–144 bp [75]. ctDNA enrichment may therefore be possible based on a size selection approach. Selecting shorter DNA fragments between 90–150 bp improved the detection of ctDNA with up to 11-fold enrichment of mutation allele fraction [61, 75, 76]. In addition, the distribution of differently sized DNA fragments has implications for disease staging as an indicator of cfDNA Integrity (cfDI). The cfDI is defined as the ratio of long (released from necrotic cells) to short (released from apoptotic cells) cfDNA fragments. cfDI is calculated by measuring long and small ALU sequence fragments (ALU_247_ and ALU_115_respectively) using qPCR [77]. Studies have shown that cancer patients have a higher cfDI compared to healthy controls or individuals with benign disease [78, 79]. Higher integrity is associated with increased levels of necrotic cell death in advanced disease with larger and more aggressive tumours [78, 80].

The short half-life of cfDNA present in the bloodstream allows for real-time analyses of the tumour mutational profile. The level of cfDNA in circulation at any given time is determined by the net amount of DNA released minus DNA clearance. cfDNA clearance may occur in organs including the liver, spleen, kidney, or lymph nodes [81]. In the bloodstream, circulating enzymes such as DNase I, plasma factor VII–activating protease (FSAP), and factor H are responsible for cfDNA breakdown [82, 83]. Rapid clearance of apoptotic cells and cfDNA normally allow for healthy individuals to have low levels of cfDNA. In cancer patients, cfDNA accumulates due to impaired clearance that is currently poorly understood. Using fetal DNA in postpartum maternal circulation, Lo et al. estimated the half-life of cfDNA to be approximately 4 to 30 min, which has been consistent across other studies [84–87]. However, the half-life of cfDNA may vary depending on several factors, including interactions with molecular complexes that interfere with cfDNA degradation, tumour stage and subtype, and treatment [69, 81]. Interestingly, one study used next-generation sequencing (NGS) technology to examine the kinetics of cfDNA and found that the clearance of cfDNA may occur in a bi-phasic manner. The first rapid phase has a mean half-life of an hour, followed by a second slow phase with a mean half-life of 13 h [88].

Several technologies have been developed for ctDNA detection in blood, including quantitative PCR, digital droplet PCR (ddPCR), and NGS for targeted sequencing or whole-genome sequencing (WGS). In addition to quantitative changes, these technologies detect qualitative changes in ctDNA, which include tumour-specific variants (TSVs), gene fusion, copy number variations, aberrant DNA methylation, and chromosomal instability. The development of NGS and digital polymerase chain reaction (dPCR) has improved the sensitivity and specificity of ctDNA detection. To date, most ctDNA detection methods have focused on high-grade serous ovarian cancer (HGSOC) patients with targeting TP53 mutations [45, 89–91]. One study used targeted error correction sequencing (TEC-Seq) to examine 58 cancer-related genes encompassing 81 kb and reported the highest sensitivity and specificity at 75–100% and > 80%, respectively [89]. In stage I-II disease, the highest detection rate was 68% with a specificity of 100% that was achieved using TEC-Seq and ddPCR combined. The high specificity achieved in this study may be attributable to TEC-Seq advantages for using deep sequencing for more direct evaluation of sequence changes. In fact, deep sequencing using random unique molecular barcodes annealed to each DNA template fragment has been the preferable method for detecting low-level signatures of TSVs in liquid biopsies [92]. Duplex sequencing using molecular barcodes on both DNA strands for removing sequencing errors that are in one strand only has improved variant detection accuracy by > 10,000 times compared to conventional NGS [93, 94].

Although ctDNA analysis with plasma samples is currently the preferred method, alternative approaches have utilized different sources. In 2013, Kinde et al. examined the ability of the liquid Pap test with uterine cervix sampling to detect ovarian and uterine cancers. The researchers used massively parallel sequencing for TSVs using a 12-gene panel and found ctDNA in 41% (9 of 22) of ovarian cancer patients [95]. Similarly in 2018, Wang et al. analyzed Pap brush samples from 245 ovarian cancer patients using PapSEEK with an assay for mutation in 18 genes and reported a limited detection sensitivity of 33%, including 34% for patients with stage I–II disease [96]. Likewise, Maritschnegg et al. conducted a study with uterine cavity lavage samples from EOC patients and benign gynecologic patients [97]. The researchers used NGS for sequencing *AKT1*, *APC*, *BRAF*, *CDKN2A*, *CTNNB1*, *EGFR*, *FBXW7*, *FGFR2*, *KRAS*, *NRAS*, *PIK3CA*, *PIK3R1*, *POLE*, *PPP2R1A*, *PTEN*, and *TP53* genes to analyze lavage samples. Using NGS, the researchers reported detectable mutations, mainly in *TP53*, in 60% of ovarian cancer patients and an improved ctDNA detection rate of up to 80% with more sensitive methods of digital droplet polymerase chain reaction (ddPCR) and the Safe-sequencing system (SafeSeqS). Interestingly, the study also found *TP53 *mutations in lavage samples of all 5 patients with stage IA disease. Building on these results, the same team conducted a study in 2018 demonstrating the feasibility of this technique that found a median absolute amount of 2.23 μg cfDNA in uterine and tubal lavage samples and TSVs using deep-sequencing in 80% (24 of 30) of ovarian cancer patients [27]. Given these findings, the molecular analysis of uterine lavage samples may be a potential technique for the early diagnosis of ovarian cancer. Other novel techniques including peritoneal washing, urine sampling, and vaginal sampling have been utilized for ctDNA profiling. However, such methods require more research to elucidate their diagnostic utility in ovarian cancer [98–101].

### cfRNA: cell-free mRNA, miRNA, circRNA and lncRNA

The rapid turnover of tumours results in high gene transcription and shedding of high amounts of cfRNA consisting of mRNA and microRNA (miRNA) into the circulation [102]. Normal and tumour cells secrete miRNAs into various body fluids, including plasma, urine and vaginal discharge, and breast milk [103]. In the blood, mRNA and miRNA are bound to specific ribonucleoprotein complexes, high-density lipoproteins, platelets, or packaged in extracellular vesicles (EV) such as exosomes to avoid degradation and acquire more stability [103, 104]. Several studies have suggested the role of miRNAs in tumorigenesis, cell differentiation, proliferation, inhibition of angiogenesis, metastasis, and apoptosis. Importantly, the biogenesis and activation of miRNAs are faster with longer half-lives compared to mRNA and proteins, which may make miRNAs more suitable for earlier diagnosis of ovarian cancer [105–108].

The diagnostic, prognostic, and therapeutic potential of circulating miRNAs in ovarian cancer have been explored in many studies. In 2008, Taylor et al. first reported that higher levels of 8 exosomal miRNAs (miR-21/141/200a/200b/200c/203/205/214) were found in the serum of ovarian patients compared to healthy controls, although there was no significant difference in early versus late-stage ovarian cancer [109]. These findings were subsequently supported by several other studies reporting that serum miRNAs (miRNA-141/200a/200b/200c) were upregulated in ovarian cancer patients compared to normal or benign tumour controls [110, 111]. Gao et al. also found that different miRNA-200c expression levels may correlate with ovarian cancer staging, with more advanced tumours having lower miRNA-200c levels and higher miRNA-141 [110]. However, Kim S. et al. analyzed seven serum exosomal miRNAs and concluded that the expression of miRNA-141, 200a, and 200b were too low to be an appropriate serologic biomarker [112].

Although miRNA-145 was identified as the best-performing single marker with a sensitivity of 91.7% and accuracy of 86.8%, similar changes in miRNA-145 levels were observed in other malignancies besides ovarian cancer [112, 113]. The lack of discrimination between cancer types suggests that single miRNAs are unlikely to be a reliable biomarker. To overcome these challenges, a recent study by Elias et al. was the first to combine NGS analysis of serum circulating miRNA with a machine learning technique called a neural network model and developed a diagnostic algorithm for EOC. The study authors reported an AUC value of 0.90 for this model, which was significantly higher compared to CA-125. This study suggests the potential for a new era of machine-learning application in biomarker discovery [114].

In 2017, Yokoi et al. performed miRNA sequencing to identify the optimal combination of candidate circulating miRNAs for the early detection of ovarian cancer [115]. This study identified eight miRNAs with RT-qPCR validation and statistical cross-validation with a large research cohort. The predictive model using a combination of 8 circulating serum miRNAs was able to differentiate early-stage ovarian cancer from benign tumours with 86% sensitivity and 83% specificity, and from healthy controls with 92% sensitivity and 91% specificity [115]. In a later study, the same research team analyzed 4,046 serum samples from 333 ovarian patients, 95 benign or borderline ovarian tumours, 2,759 healthy controls and 859 other solid cancers using miRNA microarray [116]. The study found that combined miRNAs can successfully discriminate ovarian from lung, gastric, breast, hepatic, colorectal, and pancreatic cancers, but not sarcoma or esophageal cancer. In this study, utilization of circulating miRNA yielded a sensitivity of 99% and a specificity of 100% for discriminating between ovarian cancer and healthy controls. This was the first large-scale comprehensive study examining circulating miRNAs in ovarian cancer and reported promising miRNA combinations for the detection of early-stage disease.

In addition to miRNA, circular RNAs (circRNAs) and long non-coding RNAs (lncRNAs) also demonstrated potential utility as biomarkers for liquid biopsy in ovarian cancer. circRNAs have a covalently closed loop structure and lncRNAs have transcript sizes of > 200 nucleotides, which allow increased stability and resistance against RNase degradation in the peripheral circulation. circRNAs are abundant and diverse, with a half-life > 48 h, that facilitate easier detection [117–119]. circRNA expression differs between primary and metastatic sites and is thought to play a role in regulation of ovarian cancer. A recent study found that the expression levels of circular RNAs are inversely associated with activating many signalling pathways involved in tumour metastasis (i.e., NF-κB, PI3k, AKT, and TGF-β) [120]. Using RT-qPCR in a sample of 83 EOC patients compared to 166 benign or healthy controls, Hu and colleagues found that CircBNC2 was associated with histological grade, serous subtype, and distant metastasis [121]. Similarly, lncRNAs were found to contribute to the early pathogenesis, progression, metastasis and chemoresistance of recurrent ovarian cancer [122–124]. Although there is emerging evidence suggesting an association between differing expression levels of lncRNAs (H19, LSINCT5, XIST, CCAT2, HOTAIR, AB073614, and ANRIL) and clinical progression or treatment response of ovarian cancer, the diagnostic sensitivity and specificity of lncRNAs remain to be fully elucidated [125–130]. To date, no lncRNA has been approved for clinical utility and further research is required to identify the most clinically relevant candidates with cancer-enriched or specific signatures in ovarian cancer.

### TEPs: RNA content

Tumour-educated platelets (TEPs) play an important role in local and systemic responses to tumour growth. Platelets are normally anucleate, although they may contain residual mRNA and miRNA derived from their megakaryocyte precursors or captured from intercellular interactions in the circulation. Platelet education denotes the transfer and sequestration of biomolecules from tumour cells into platelets [131, 132]. External factors in the tumour microenvironment such as stromal and immune cell signals may activate platelet surface receptors to induce specific splice events of pre-messenger RNAs (pre-mRNAs) in circulating platelets [133, 134]. Key advantages of TEPs include their high abundance, easy isolation, and high-quality RNA that may be processed according to external signals. Therefore, TEPs have a dynamic mRNA repertoire with both specific splice events in response to external signals and direct ingestion of spliced circulating mRNA that may provide useful diagnostic information in ovarian cancer. Best et al. first studied the diagnostic potential of TEPs by mRNA sequencing in patients with various cancers [135]. This study found that TEPs were able to distinguish cancer patients from healthy controls with a high accuracy of up to 96% and detect the primary tumour location with 71% accuracy. Later, Piek et al. concluded that TEPs can differentiate early stage ovarian cancer from benign pathologies with 80% accuracy [136]. An ongoing clinical trial (NCT04022863) may further build upon these results by examining the accuracy of TEPs and ctDNA in determining the nature of ovarian tumours and provide information on its diagnostic potential [137]. Interestingly, a recent retrospective cohort study by Giannakeas et al. examined the association between thrombocytosis (platelet count greater than 450 × 109/L) and cancer. By studying 53,339 adults aged 40–75 years who developed thrombocytosis with normal platelet count in the previous 2 years and no malignancy history, they estimated the risk of cancer in a 10-year follow-up period [138]. The authors reported that the 2-year relative risk (RR) was highest for ovarian cancer (RR = 7.11; 95% CI, 5.59–9.03), while the 6-month RR for developing ovarian cancer was even higher (RR = 23.33; 95% CI, 15.73–34.61). In the future, TEPs profiling with complementary ctDNA/CTC analysis and platelets quantification may potentially be a blood-based method for cancer diagnostics.

### Exosomes: content analysis

Interest in the diagnostic and prognostic potential of exosomes has increased in recent years. Exosomes are extracellular vesicles (EVs) typically 30–100 nm in diameter. Such vesicles are extremely stable and can survive under extreme conditions. Exosomes are released from both normal and tumour cells. Likewise, they are found in various body fluids, such as saliva, plasma, urine, ascites, and cerebrospinal fluid [139]. Exosomes can participate in local and distant signalling by fusing with the membrane of the recipient cell or attaching to receptors on the cell’s surface. In cancer, exosomes have the ability to enhance tumorigenesis [140], help tumour cells escape the immune system [141], and cause treatment resistance [142]. Likewise, exosomes can enter the circulation and increase the likelihood of metastasis by preparing distant tumour microenvironments [143, 144]. Exosomes have been also used as therapeutics to successfully eliminate tumour cells [145]. Furthermore, exosomes contain tumour-specific proteins, lipids, DNA, and RNA making them potential diagnostic biomarkers in cancer. For example, exosomes with heat shock protein (HSP70) expressed on their membrane, are observed more in ovarian, breast, and lung cancer samples compared to healthy controls [146]. Additionally, studies have shown increased total exosome concentrations in serum samples of EOC patients [109, 111].

Exosomes can carry significant quantities of miRNAs. Multiple studies have observed differences between the miRNA profiles of exosomes in EOC patients and healthy controls. Meng et al. showed that the concentrations of miR-200b and miR-200c are higher in exosomes obtained from patients with stage III–IV EOC and are associated with significantly shorter OS [111]. Another study showed that the miRNA profiling of circulating exosomes using a modified magnetic-activated cell sorting (MACS) technique can differentiate between benign and malignant ovarian tumours [109]. Additionally, exosomes derived from EOC patients have higher concentrations of TGFB1 and melanoma-associated antigen 3 (MAGE3) and MAGE6 [147]. EOC exosomes also have a higher concentration of Claudin 4 that is associated with tumour stage and CA125 levels [148]. CD24 and EpCAM were also shown to be elevated in exosomes isolated from EOC plasma samples [149]. Furthermore, Liang et al. identified 2,230 proteins in exosomes secreted from OVCAR-3 and IGROV1 ovarian cancer cell lines. Many of these identified proteins were involved in tumorigenesis and metastasis, indicating the prognostic potential of exosomal profiling [150]. Overall, exosomal profiling can act as a cancer-specific diagnostic and prognostic biomarker and replace invasive cell biopsies. However, more comprehensive clinical studies are required to determine the clinical value of this approach.

## Clinical applications of liquid biopsy for ovarian cancer

**Fig. 3:**
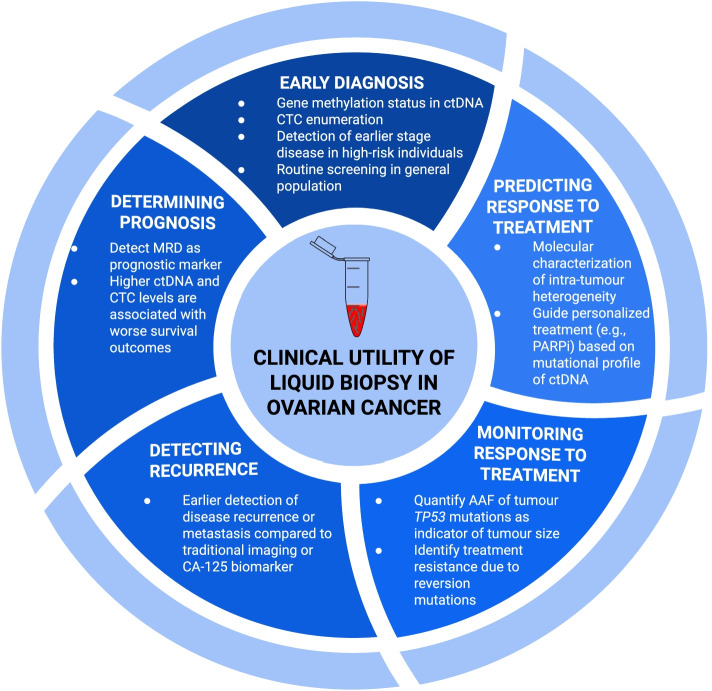
Overview of the five major clinical applications of liquid biopsy in ovarian cancer. Earlier in the disease course, sample analysis for ovarian cancer biomarkers can allow earlier diagnosis. Following primary debulking surgery, liquid biopsy can detect minimal residual disease as a prognostic indicator and allow for earlier detection of recurrent disease. During treatment, liquid biopsy may enhance longitudinal monitoring with its non-invasive approach that enables serial sampling. Additionally, liquid biopsy offers the advantage of capturing the entire tumour genome, which can help identify novel genetic markers for targeted therapies and detect treatment resistance. ctDNA: circulating tumour DNA, MRD: minimal residual disease; AAF: alternative allele frequency

**Fig. 4:**
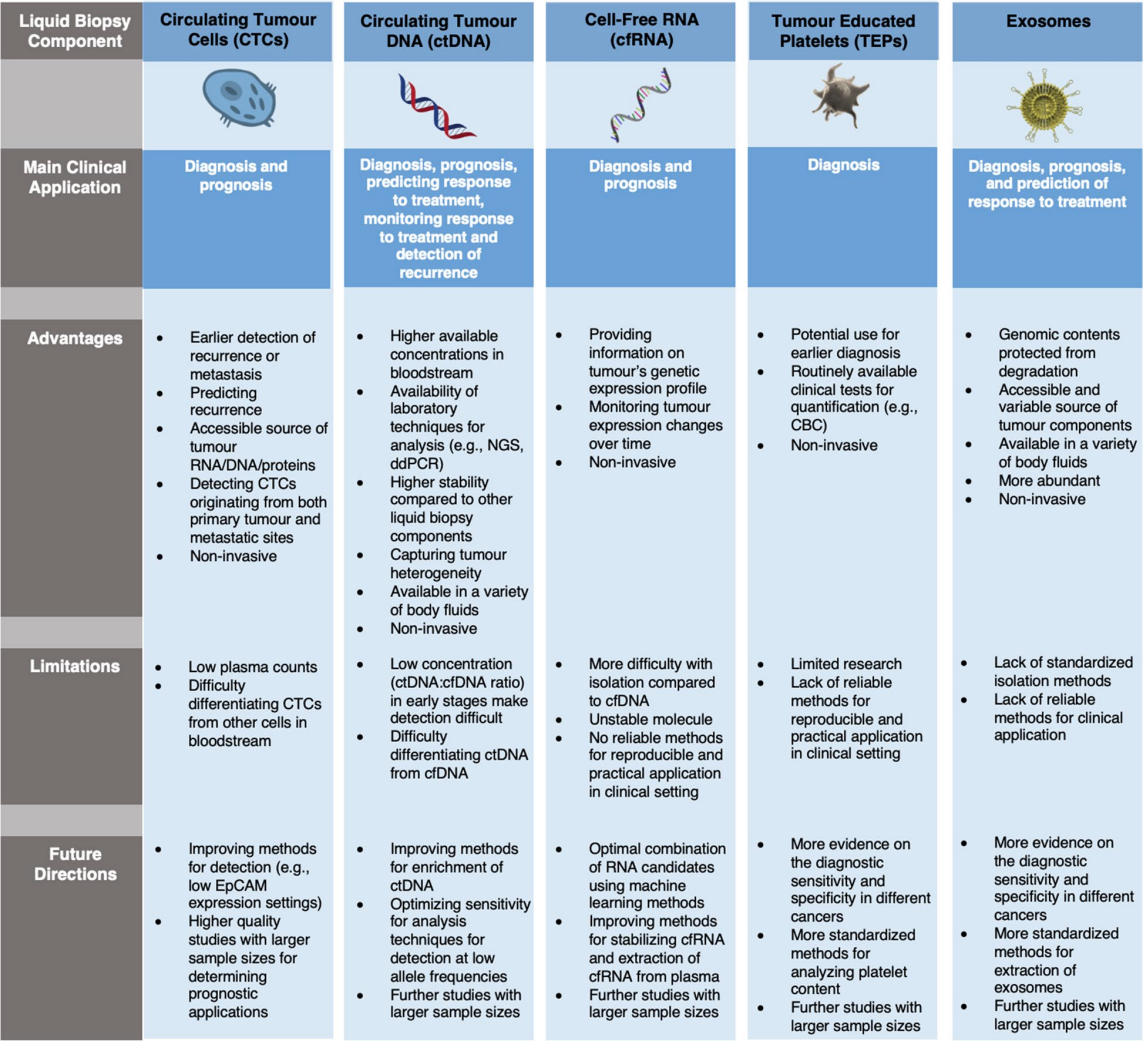
Comparison of five liquid biopsy components and the main advantages, disadvantages, and future directions of their clinical application in ovarian cancer management

Liquid biopsy is a non-invasive method with emerging evidence for its utility in screening and longitudinal monitoring of ovarian cancer. Different tumour components may be analyzed within collected plasma samples to provide earlier diagnosis, prognostication, molecular targets for therapy, and detection of resistance to treatment (Fig. 3). A summary of the key advantages, disadvantages and main clinical applications of each liquid biopsy component is provided in Fig. 4.

### Early diagnosis of ovarian cancer

The early detection of ovarian cancer is critical in reducing mortality and morbidity. Staging is the most important prognostic factor in determining recurrence-free survival (RFS), with stage I or II diseases having significantly longer RFS and OS compared to more advanced stages [151]. Therefore, biomarkers that allow for the diagnosis of OC in stages I-II may extend survival and improve patient outcomes.

Two-thirds of EOC cases are diagnosed at advanced stages, with a significantly worse prognosis [152]. Interestingly, several studies have suggested the diagnostic potential of promoter methylation that leads to epigenetic inactivation of tumour suppressor genes as an early event during ovarian cancer pathogenesis [153–160]. Analysis of ctDNA methylation status in pre-operative plasma samples from ovarian cancer patients with both early (stage I-II) and advanced-stage disease (stage III-IV) demonstrated a significant association with abnormal methylation of tumour suppressor genes compared to healthy controls [154, 157, 158]. Specifically, the detection of hypermethylation in promoter regions of tumour suppressor genes *RUNX3, TFPI2, RASSF1* and *RASSF2 *from plasma samples has been reported for the diagnosis of ovarian cancer, although these markers are also implicated in other cancers [153–160]. Despite the potential for identifying aberrant gene promoter methylation to detect malignancies, its utility remains limited by the concentration of extracted ctDNA available for analysis. Earlier stages of EOC are often asymptomatic and are correlated with lower concentrations of ctDNA [159, 160]. There is currently limited evidence regarding the application of liquid biopsy for the early detection of ovarian cancer at pre-symptomatic stages. Only one study by Widschwendter et al. conducted in the early screening setting reported that methylation in a set of epigenetic markers including *COL23A1*, *C2CD4D* and *WNT6*is able to detect EOC up to 2 years before clinical diagnosis with a sensitivity of 23% and specificity of 97% [161]. This study used reduced representation bisulfite sequencing (RRBS) to analyze samples collected from 100,000 asymptomatic women and 43 cases of ovarian cancer. It remains difficult to conduct screening studies for EOC due to the relatively low incidence and large sample sizes required.

Other approach is looking for gene mutations rather than methylation changes. The sensitivity and specificity of mutation detection are important considerations when applying liquid biopsies for the early diagnosis of ovarian cancer. ctDNA has better diagnostic performance compared to traditional CA-125 with several studies demonstrating that quantitative analysis of ctDNA has a relatively high specificity of 88% and sensitivity ranging from 27–100% [92, 162–168]. A recent systematic review of 23 studies evaluating ctDNA for the diagnosis of EOC in symptomatic patients preoperatively yielded similar results [89, 96, 153, 154, 156–158, 160–162, 166, 167, 169–179]. Other studies that evaluated ctDNA analysis in HGSOC patients for *TP53*mutation detection, reported high sensitivity (75–100%) and specificity (> 80%) [45, 89–91]. For stage I-II disease, Phallen et al. observed a sensitivity of 68% and a specificity of 100%. The researchers used TEC-Seq and ddPCR for ctDNA detection and found detectable mutations in driver genes for over two-thirds of stage I-II ovarian cancer cases [89]. More recently, Barbosa et. al used NGS and a custom panel of 27 genes to analyze tumour and matched plasma samples from 96 ovarian cancer patients and detected tumour somatic variants in 75% of patients with stage I disease [180]. Cohen et al. analyzed circulating protein biomarkers and genetic alterations in cfDNA using a commercial blood test called CancerSEEK [177]. This test used 61 amplicons for massively parallel sequencing to increase sensitivity while minimizing any changes in specificity. The authors reported that ctDNA detected in ovarian cancer with a sensitivity of 98% and specificity of 99%, however, the early-stage detection rate was only 38% [177].

CTC is another liquid biopsy component that has been studied for early EOC detection. Zhang et. al used immunomagnetic bead screening, targeting epithelial markers EpCAM, HER2, and MUC1 on ovarian cancer cells, combined with multiplex RT-qPCR analysis of isolated mRNA from CTCs and tumour tissues for detecting CTCs in 109 EOC patients. This study showed that patients with stage IA-IB disease have a much higher CTC positive rate (93%) compared to the CA-125 positive rate (64%) in the same patients [181]. In 2018, Guo et al. prospectively enrolled 61 women with suspicious ovarian masses to investigate the diagnostic value of CTCs. The researchers used microfluidic isolation and immunofluorescent staining to identify and quantify CTCs and determined the sensitivity of CTCs using receiver operating characteristic (ROC) curve analysis to be 73.3% with a specificity of 86.7%, which was superior to CA-125 (sensitivity = 56.7%) [182]. The main challenge facing CTC-based diagnostics is the reduced amount of CTCs detectable in circulation with lower tumour burdens at early disease stages [15, 182, 183]. In contrast, advanced stage tumours release more CTCs that often travel with metastatic colonies to distant sites allowing for easier detection. As such, detection of CTCs is significantly associated with advanced stages (III and IV), where the diagnostic performance of CTCs through immunocytochemistry (ICC) has been reported to have a sensitivity of 76–83% and specificity of 55–95% [21, 25, 181, 184, 185]. Compared to benign controls, early-stage (I-II) and late-stage (III-IV) EOC samples are 8.4 and 16.9 times more likely to have CTCs, respectively. Likewise, significantly lower levels of CTCs are found in stage I patients compared to advanced stages [181]. Current studies on utilizing CTC for early diagnosis of ovarian cancer are limited by small sample sizes and future well-powered studies are warranted to confirm these findings.

Although some studies have suggested the diagnostic potential of miRNA expression profiling and exosome analysis in ovarian cancer, further research is required to determine their diagnostic sensitivity and specificity in early-stage disease. For example, Todeschini et al. analyzed two cohorts consisting of 168 stage III-IV HGSOC patients and 65 healthy controls and demonstrated the clinical potential of miR-1246 as a diagnostic biomarker for HGSOC with a significant increase in the expression of miR-1246 in post-operative serum samples of HGSOC compared to healthy individuals. This study used a novel microarray data normalization to identify candidate diagnostic miRNAs followed by signature validation with RT-qPCR. The study reported a significant over-expression of miR-1246, miR-595, and miR-2278 in HGSOC patients and the highest detection performance for miR-1246, with a diagnostic sensitivity of 87%, specificity of 77%, accuracy of 84%, and AUC of 0.89 [184]. Similarly, the clinical potential of exosomes have also been suggested in ovarian cancer. Zhang et. al analyzed plasma samples from 40 stage III or IV EOC patients versus 40 healthy controls to investigate the role of four exosome proteins including Lipopolysaccharide Binding Protein (LPB), Fibrinogen Gamma Chain (FGG), Fibrinogen Alpha Chain (FGA) and Gelsolin (GSN) as diagnostic biomarkers [186]. This study reported significantly elevated FGA and GSN levels and significantly downregulated FGG and LBP levels in the ovarian cancer group. FGA conferred the highest diagnostic sensitivity among the 4 candidates with an AUC of 0.8459. In another study with 78 EOC patients (63 stage III-IV and 7 stage I-II) and 30 healthy controls, Schwich et al. reported a seven-fold increase in HLA-G levels in plasma circulating exosomes of EOC patients (mean 14.3 ng/mL) compared to healthy controls (1.9 ng/mL) [187]. Therefore, studies to date on miRNA and exosome analysis have been conducted mainly in patients with advanced ovarian cancer, and future research is needed to elucidate their diagnostic utility for early-stage disease. Table 1 summarizes the studies investigating the clinical application of liquid biopsy analytes in ovarian cancer diagnosis.

**Table 1:** Liquid biopsy analytes and potential utility as diagnostic biomarkers

Analyte	Author, Year	Tumour Subtype and Staging	Number of patients	Laboratory Technique	Detection Rate	Ref
CTCs	Zhang et al., 2018	Stage I-IV EOC	109	Immunomagnetic bead screening, Multiplex RT-PCR	90%	[181]
	Guo et al., 2018	Stage I-IV EOC	30	Microfluidic isolation and immunofluorescent staining	73%	[182]
	Pearl et al., 2014	Stage I-IV EOC	129	CAM-based identification platform	Sensitivity = 83% PPV = 97.3%	[184]
	Poveda et al., 2011	Stage I-IV EOC	216	CellSearch system and reagents (Veridex)	14.4% had 2 or more CTCs prior to therapy	[183]
	Pearl et al., 2015	Stage I-IV EOC	123	iCTC flow cytometry assay	Sensitivity = 83% Specificity = 97%	[185]
ctDNA	Wang et al., 2017	Stage I-IV EOC	194	QIAamp DNA blood mini kit, promoter methylation OPCML, TFPI2 and RUNX3	Sensitivity = 90.14 Specificity = 91.87	[154]
	Dong et al., 2012	Stage I-IV EOC	36	Methylation-specific PCR	80.6%	[158]
	Wu et al., 2014	Stage I-IV EOC	47	Methylation-specific PCR	51.1%	[156]
	Bondurant et al., 2011	Stage I-IV EOC	106	Methylation-specific PCR	51%	[159]
	Liggett et al., 2011	Stage III-IV EOC	30	Microarray-mediated methylation assay	Sensitivity = 90.0% Specificity = 86.7%	[160]
	Widschwendter et al., 2017	Stage I-IV EOC	43	Reduced representation bisulfite sequencing	Sensitivity = 23% Specificity = 97%	[161]
	Forshew et al., 2012	Stage III-IV EOC	46	Targeted deep sequencing	Sensitivity = 97.5% Specificity = 97.5%	[162]
	Du et al., 2018	Stage II-III EOC	21	High-throughput sequencing	Sensitivity = 73.7% Specificity = 100%	[165]
	Vanderstichele et al., 2017	Stage I-IV EOC	57	Whole-genome sequencing	Sensitivity = 2- to fivefold higher than CA-125 Specificity = 99.6%	[166]
	Cohen et al., 2016	Stage I-IV EOC	32	DNA sequencing and whole genome NIPT	Sensitivity = 40.6% Specificity = 93.8%,	[167]
	Wang et al., 2015	Stage I-IV EOC	114	Multiplex nested methylated specific PCR	Sensitivity = 90.14% Specificity = 91.06%	[155]
	Zhang et al., 2013	Stage I-IV EOC	87	Methylation-specific PCR	Sensitivity = 89.66% Specificity = 90.57%	[157]
	Dvorská et al., 2019	Stage I-IV EOC	49	Pyrosequencing	Sensitivity = 98% Specificity = 56%	[171]
	Su et al., 2009	Stage I-IV EOC	26	Methylation-specific PCR	Sensitivity = 73% Specificity = 75%	[172]
	Melnikov et al., 2009	Stage I-IV EOC	33	Microarray mediated methylation assay	Sensitivity = 85% Specificity = 61%	[175]
	Singh et al., 2020	Stage I-IV EOC	70	TaqMan based qPCR assay	Sensitivity = 89% Specificity = 100%	[176]
	Cohen et al., 2018	Stage I-III EOC	54	Combined assays for genetic alterations and protein biomarkers (CancerSEEK)	Sensitivity = 98% Specificity = 99%	[177]
Exosomes	Schwich et al., 2019	Stage I-IV EOC	78	Nanoparticle tracking analysis, ELISA	100%	[187]

### Detecting recurrence and determining prognosis in ovarian cancer

There is ongoing research examining the clinical application of liquid biopsy to identify microscopic residual disease following primary debulking surgery as a prognostic indicator, predict survival outcomes, and for earlier detection of disease recurrence. Clinically, the implementation of liquid biopsy may aid in selecting individuals at greater risk of relapse for consideration of alternative management approaches and potential inclusion in clinical trials.

The strongest evidence to date on the prognostic utility of liquid biopsy is for ctDNA [188, 189]. Quantitative analysis of ctDNA demonstrate that ctDNA concentrations are positively correlated with advanced stages of EOC and may indicate response to treatment [188–192]. ctDNA concentrations are more associated with earlier progression and decreased response to treatment than CA-125 or imaging [162–164, 193]. Pereira et al. used qPCR and targeted sequencing to quantify ctDNA levels immediately following surgery in 22 EOC patients. This study found that undetectable levels of ctDNA at 6 months postoperatively were associated with significantly improved PFS (*P* = 0.001) and OS (*P*< 0.05) [163]. Another study examining *TP53* mutations in ctDNA of relapsed HGSOC patients highlighted the prognostic ability of *TP53*, with a less than 60% decrease in *TP53 *mutant allele fraction after one cycle of chemotherapy associated with poor response and shorter PFS compared to a decrease of more than 60% [164]. Similarly, a recent study with 48 HGSOC patients found that approximately 80% of patients classified as having no surgical residual disease had detectable ctDNA. This study reported that these patients had a higher mortality risk compared to those who did not have detectable post-surgery ctDNA with a 5-year survival rate of 58.3% for those with detectable ctDNA, compared to 85.7% for those with undetectable ctDNA [193]. Although most studies were limited by small sample sizes, all authors concluded that analyzing ctDNA through liquid biopsy has potential as a prognostic biomarker in clinical settings.

Furthermore, there is also evidence supporting the role of ctDNA in detecting recurrent disease. Up to 85% of EOC patients can experience recurrence following first-line therapy. Recurrence of EOC limits the survival of patients and is often referred to as incurable. CA-125 and imaging techniques such as CT and PET-scans are used as traditional recurrence markers [194]. However, recent studies have shown that ctDNA quantification can potentially improve the detection of relapse compared to traditional imaging techniques and CA-125 [188, 190]. Similarly, Parkinson et al. examined TP53 mutations in ctDNA of relapsed HGSOC patients and reported that ctDNA was detected at ≥ 20 amplifiable copies/mL of plasma in nearly all relapsed patients with disease volume > 32 cm^3^ [164]. Likewise, Minato et al. detected ctDNA in all patients with recurrent EOC using droplet digital PCR, while no ctDNA was detected in recurrence-free patients. In the majority of cases, ctDNA was detected before CA-125 levels indicated recurrence [195]. These results are consistent with the study by Pereira et al. that reported a mean predictive lead time of 7 months for ctDNA over CT imaging for the detection of recurrence [163]. As a result, ctDNA has the potential to be used as an early detection tool for EOC recurrence.

CTCs have also demonstrated prognostic potential in ovarian cancer. In a study by Marth et al., immunobeads coated with MOC-31 antibodies were used to isolate CTCs from blood samples of 90 EOC patients. The authors did not find an association between the detection of CTCs and poor prognosis [18]. Using the CellSearch system, Poveda et al. found that among 216 ovarian cancer patients after primary debulking surgery and diagnosis of recurrence with failed first-line chemotherapy, those with higher CTC levels, defined as ≥ 2 CTCs per 7.5 mL blood, had a 2.06-fold (*p* = 0.003) higher overall mortality risk and 1.89-fold (*p*= 0.003) higher risk of progression on doxorubicin treatment [183]. On the contrary, a multivariate analysis conducted by Marth et. al reported no statistically significant correlation between the presence of CTCs in the bloodstream before surgery and PFS or OS. This study reported a mean overall survival of 25 and 28 months for patients with and without detected CTCs, respectively [18]. Similarly, Judson et al. analyzed pre-operative blood samples from 64 EOC patients and reported no significant difference in the OS (*p* = 0.96) or PFS (*p*= 0.72) between patients with and without detectable CTCs at a mean follow-up of 18.7 months [196]. Given this controversy, Huang et al. subsequently conducted a meta-analysis including 1285 patients from 2 clinical trials and 13 retrospective studies that demonstrated a significant association between the presence of CTCs before treatment with surgery or chemotherapy and both OS (HR = 1.79, 95% CI:1.43–2.24, *p* < 0.00001) and PFS (HR = 1.59, 95%CI:1.30–1.94, *p *< 0.00001) [197]. Other studies have suggested that the number of CTCs may be a potential prognostic factor for EOC patients, but the results are limited by small sample sizes and contradictory findings [21, 181–183, 198–201]. For example, some studies highlighted the prognostic role of CTCs, showing that the amount of pre-operative or post-adjuvant chemotherapy CTCs was associated with poor prognosis reflected in both PFS or OS [21, 181, 183, 199–201]. However, the results of these studies are inconsistent with other investigations that failed to find similar associations [22, 182, 198].

Currently, the prognostic value of other liquid biopsy components including cell-free miRNAs and exosomes lacks enough evidence for clinical applications. Several limiting factors in cell-free miRNAs studies, including the lack of standardized experimental procedures, varied normalization processes, and the inadequately powered sample sizes for statistical analysis have contributed to the controversy in this domain [110, 111, 186, 202, 203]. Several studies have supported the prognostic role of cell-free miRNAs such as miR-200 family, particularly miR-200a, miR-200b, and miR-200c [110, 111, 202, 203]. In another study with 40 EOC patients, Zhang et al. used Western blot analysis and enzyme-linked immunosorbent assay (ELISA) to analyze exosomal protein markers [186]. The study reported high levels of fibrinogen gamma chain (FGG) or lipopolysaccharide binding protein (LBP) mRNA expression were associated with worse prognosis and shorter PFS and OS for patients with EOC (FGG: OS HR = 0.79, *P* = 0.001 and PFS HR = 0.77, *P* < 0.001 for LBP: OS HR = 0.81, *P* = 0.003 and PFS HR = 0.77, *P*< 0.001) [186]. Since EVs contain several different tumour-derived components, EVs may be a promising all-in-one prognostic biomarker, providing information on the tumour and its microenvironment. However, the absence of a standardized approach for cell-free miRNA and EVs isolation and the small sample sizes of current studies limit the ability to draw definitive conclusions and require future validation in larger cohorts. Table 2 summarizes the studies investigating the clinical application of liquid biopsy analytes as prognostic biomarkers in ovarian cancer.

**Table 2:** Liquid biopsy analytes and potential utility as prognostic biomarkers

Analyte	Author, Year	Tumour Subtype and Staging	Number of patients	Laboratory Technique	Prognostic Significance	Ref
CTCs	Zhang et al., 2018	Stage I-IV EOC	109	Immunomagnetic bead screening, RT-PCR	OS (*p* = 0.041)	[181]
	Poveda et al., 2011	Stage I-IV EOC	216	CellSearch system and reagents	OS (*p* = 0.0017) PFS (*p* = 0.00024)	[183]
	Judson et al., 2003	Stage I-IV EOC	64	Immunocytochemical assay	NS	[196]
	Aktas et al., 2011	Stage I-IV EOC	122	AdnaTest BreastCancer, RT-PCR	OS (*p* = 0.0054)	[199]
	Chebouti et al., 2017	Stage I-IV EOC	65	AdnaTest Ovarian Cancer, RT-PCR	OS (*p* = 0.0008) PFS (*p* = 0.0293)	[200]
	Kuhlamann et al., 2014	Stage I-IV EOC	143	Multiplex RT-PCR, immunomagnetic CTC enrichment	OS (*p* = 0.026) PFS (*p* = 0.009)	[16]
	Obermayr et al., 2013	Stage I-IV EOC	216	RT-qPCR, microarray analysis	OS (*p* = 0.001) PFS (*p* = 0.001)	[21]
	Obermayr et al., 2017	Stage I-IV EOC	266	Density gradient centrifugation, immunostaining, FISH	OS (*p* = 0.007) PFS (*p* = 0.008)	[201]
ctDNA	Giannopoulou et al., 2017	Stage I-IV EOC	59	Methylation-sensitive high-resolution melting analysis (MS-HRMA) assay	OS (*p* = 0.023)	[153]
	Pereira et al., 2015	Stage I-IV EOC	10	Droplet digital PCR	OS (*p* = 0.0011) PFS (*p* = 0.0194)	[163]
	Parkinson et al., 2016	Stage I-IV EOC	40	Microfluidic digital PCR	TTP (*p* = 0.008)	[164]
	Swisher et al., 2005	Stage I-IV EOC	137	DNA sequencing, PCR	OS (*p* = 0.02)	[58]
	Giannopoulou et al., 2018	Stage I-IV EOC	53	Methylation-specific PCR	OS (*p* = 0.027) PFS (*p* = 0.041)	[170]
	No et al., 2012	Stage I-IV EOC	36	Copy number assay, qPCR	OS (HR = 33.6, 95% CI = 1.8–634.8) DFS (HR = 18.2, 95% CI = 2.0–170.0)	[178]
	Kuhlmann et al., 2012	Stage I-IV EOC	63	PCR-based fluorescence microsatellite analysis	OS (*p* = 0.030)	[179]
	Pearl et al., 2014	Stage I-IV EOC	129	CAM-based identification platform	CTCs were better correlated with worse OS and PFS compared to CA125	[184]
	Pearl et al., 2015	Stage I-IV EOC	123	iCTC flow cytometry assay	CTCs more sensitive to progressive disease and relapse compared to CA125	[185]
	Minato et al., 2021	Stage I-IV EOC	11	Droplet digital PCR	Earlier recurrence detection compared to CA125	[195]
	Kim et al., 2019	Stage II-IV EOC	61	Droplet digital PCR	TTP (*p* = 0.038)	[189]
	Paracchini et al., 2020	Stage III-IV EOC	46	Shallow whole-genome sequencing	PFS (*p* = 0.011)	[204]
cfRNA	Zuberi et al., 2015	Stage I-IV EOC	70	Trizol method	Disease progression (*p* = 0.001)	[189]
	Halvorsen et al., 2017	Stage I-IV EOC	207	TaqMan Low Density Arrays, RT-qPCR	OS (*p* = 0.012) PFS (*p* = 0.006)	[203]
	Zhang et al., 2019	Stage I-IV EOC	40	liquid chromatography tandem mass spectrometry	OS (*p* = 0.0012) PFS (*p* = 0.00038)	[186]
Exosomes	Schwich et al., 2019	Stage I-IV EOC	78	Nanoparticle tracking analysis, ELISA	PFS (*p* = 0.029)	[187]

*OS *Overall Survival, *PFS* Progression-Free Survival, *NS *Not Significant, *TTP *Time to Progression

### Predicting and monitoring response to treatment

**Fig. 5:**
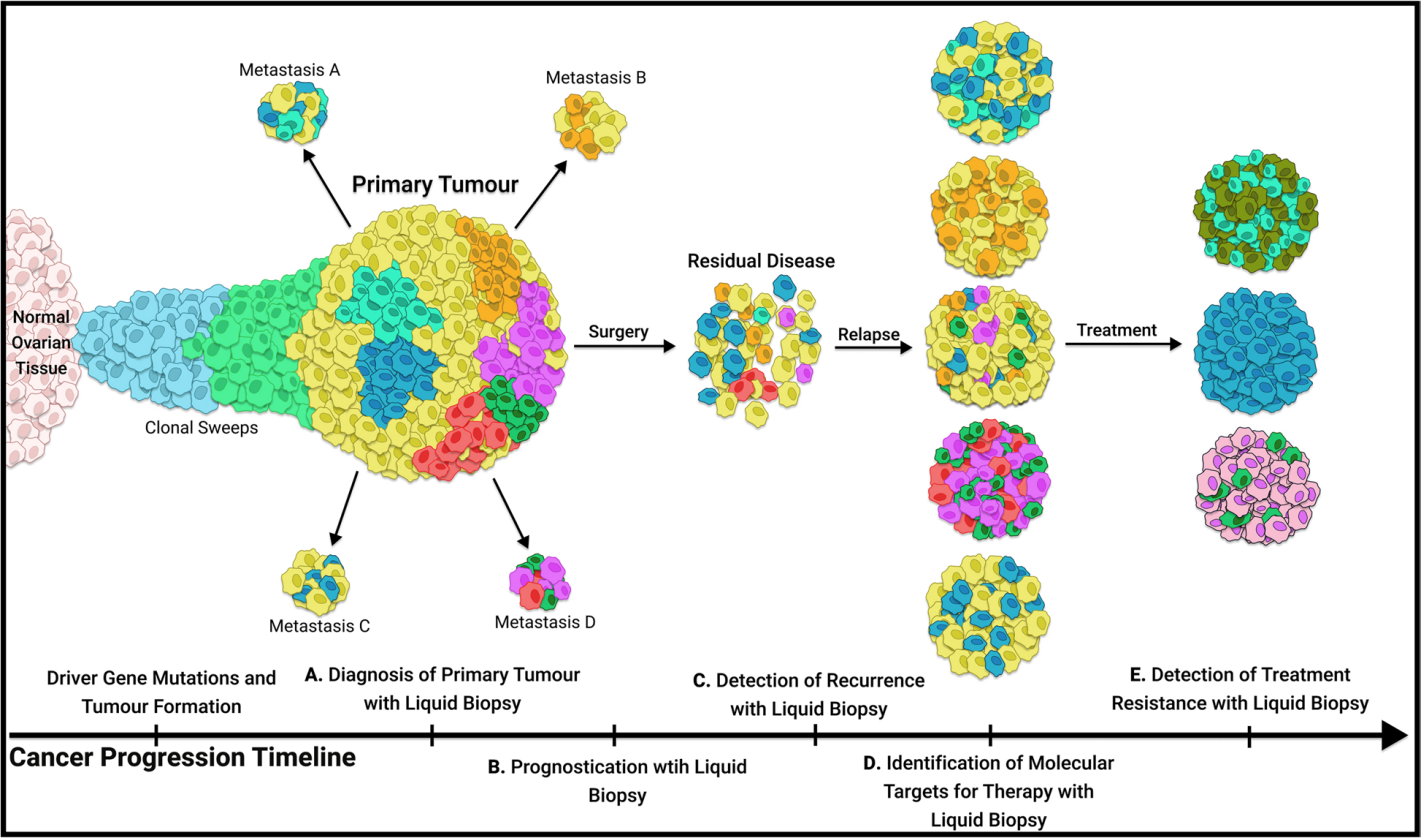
The utility of liquid biopsy during different stages of tumour progression. The molecular profile of the primary tumour changes as cancer progresses. New mutations and treatments can lead to intra-tumour heterogeneity. Furthermore, heterogeneity causes drug resistance and treatment failure. Liquid biopsy can aid in the detection of primary ovarian tumours (A). The prognosis of EOC patients can potentially be determined by liquid biopsy (B). Likewise, this technique can help with the detection of residual disease after primary debulking surgery and contribute to the detection of EOC recurrence (C). Physicians can potentially use liquid biopsy to uncover the molecular profile of the tumor and select the correct therapy for each patient (D). Liquid biopsy can also reflect tumour heterogeneity and predict resistance to platinum-based chemotherapy in addition to both primary and acquired resistance to PARPi (E)

Although most ovarian cancer patients achieve complete remission after primary debulking surgery and adjuvant chemotherapy, up to 70% of patients develop recurrence due to chemoresistance. Intra-tumour heterogeneity has been proposed as the main cause of drug resistance and treatment failure in ovarian cancer [205]. Intra-tumour heterogeneity refers to the genomic variations within a lesion that arise from tumour cell evolution during the multistep tumorigenesis process. Clonal development from a single malignant cell into a functionally heterogeneous tumour mass is shaped by the tumour microenvironment and adaptation to various external selection pressures (e.g. evasion of apoptosis, self-sufficient cell proliferation, acquisition of replicative immortality). Subclones may evolve and expand in a sequentially linear fashion, or they may follow branched trajectories by continuing to diverge during their evolution trajectory (Fig. 5) [206, 207]. The molecular characterization of all ovarian cancer subclones is crucial for selecting appropriate targeted therapy and identifying acquired resistance in tumour cell clones over time. Liquid biopsy can potentially offer more comprehensive analysis of tumour heterogeneity and allow longitudinal monitoring of tumour evolution over the course of treatment.

The ability of ctDNA to reflect tumour heterogeneity is useful for predicting resistance to platinum-based chemotherapy, in addition to both primary and acquired resistance to PARPi. Approximately half of HGSOC patients have a Homologous Recombination Repair (HRR) deficiency leading to an impaired ability to repair double-stranded DNA breaks and increased sensitivity to the alkylating action of platinum-based chemotherapies that induce DNA damage [208]. *BRCA* mutated cells also depend on single-stranded DNA repair mechanisms involving Poly ADP-ribose Polymerase (PARP), which results in susceptibility to the synthetic lethality of PARPi [209]. Although ovarian tumours with pathogenic *BRCA1/2* variants have demonstrated increased sensitivity to platinum-based chemotherapy and PARPi, the majority will subsequently develop treatment resistance. Mechanisms leading to treatment resistance may be categorized broadly into two types. One of these is the small subclonal tumour cells that do not have the *BRCA1/2 *mutations, and after starting treatment, become the main clone that does not respond to targeted treatment. The second mechanism is through acquired reversion mutations located close to the initial loss-of-function variant that effectively restore functional protein production [210, 211].

Longitudinal monitoring of *BRCA* mutation evolution in HGSOC patients during PARPi therapy may be achieved with serial cfDNA sampling. Previous studies have demonstrated that both germline and somatic *BRCA *reversion mutations can be detected in the cfDNA of patients with HGSOC [212, 213]. In the ARIEL2 trial, Lin et. al assessed the association between *BRCA* reversion mutations and clinical efficacy of adjuvant PARPi treatment. This study included 112 HGSOC patients with germline or somatic *BRCA1/2* mutations and used NGS to analyze plasma cfDNA collected prior to PARPi treatment with rucaparib after disease progression on platinum-based chemotherapy. The researchers found that the absence of reverse *BRCA* mutations in pre-treatment cfDNA was associated with a longer PFS on rucaparib with a median PFS of 9.0 months, compared to 1.8 months in patients with detectable *BRCA* reversion mutations in their cfDNA (HR 0.12; *p* < 0.0001). In addition, this study also sequenced 78 post-progression cfDNA samples to examine acquired resistance and identified an additional 8 patients with novel *BRCA *reversion mutations not initially found in pre-treatment cfDNA, suggesting the ability of cfDNA to monitor dynamic changes in *BRCA* mutational status over time [212]. Other studies have reported a similar association between detection of *BRCA1/2* reversion mutations in plasma ctDNA and resistance to adjuvant platinum and PARPi therapy (Table 3) [213, 214]. Christie et al. conducted a prospective study with 30 HGSOC patients with a germline *BRCA1/2* mutation to determine whether cfDNA analysis could predict chemotherapy response in both the neoadjuvant and adjuvant setting. In this study, paired tumour and plasma samples were collected for two cohorts of patients: one cohort comprised 14 patients receiving treatment prior to primary debulking surgery and the second cohort included 16 patients receiving treatment after recurrence with previous adjuvant platinum-based chemotherapy. The researchers used targeted NGS to first screen tumour samples for germline *BRCA1/2* mutation or reversion status before analyzing cfDNA. This study only detected *BRCA1/2* reversion mutations in tumour samples of 31.3% (5 of 16) patients in the second cohort with recurrent disease, among which 18.8% (3 of 5 patients) also had detectable reversions in cfDNA. Clinically, all cases with detectable reversions in cfDNA subsequently became resistant to platinum or PARPi therapy. Routine monitoring through liquid biopsy may therefore allow for earlier detection of resistance and selection of more personalized combination therapies (i.e. alternative chemotherapies, targeted therapies or immunotherapy) targeting different oncogenic drivers to reduce the risk of resistance.

**Table 3:** Clinical utility of ctDNA as liquid biopsy for predicting and monitoring response to treatment

Author, Year	Tumour Classification	Sample Size	Laboratory Methodology	Detected Abnormality	Treatment Protocol	Clinical Application	Ref
Gifford et. al, 2004	Stage Ic-IV EOC	138	Microsatellite PCR	hMLH1 methylation	Paclitaxel/docetaxel + carboplatin	Response monitoring	[215]
Swisher et al., 2005	Stage I-IV EOC	137	DNA sequencing	p53 mutation	Taxane + platinum agent	Response monitoring	[58]
Capizzi et al., 2008	Stage III-IV EOC	22	RT-PCR	Serum level	Carboplatin + paclitaxel or carboplatin only	Response monitoring	[216]
Kamat et al., 2010	Stage I-IV EOC	164	RT-PCR	Beta-globin	Platinum agent	Response monitoring	[217]
Wimberger et al., 2011	Stage Ib-IV EOC	62	Fluorescence	Fluorimetry	Carboplatin + paclitaxel	Response monitoring	[218]
Forshew et al., 2012	HGSOC	38	TAm-Seq, dPCR	*TP53, PTEN, BRAF, KRAS, EGFR, PIK3CA* mutations	Carboplatin + paclitaxel or epirubicin + cisplatin + capecitabine	Response monitoring	[162]
Murtaza et al., 2013	Stage III-IV	3	NGS, qPCR	*RB1, ZEB2, BUB1, CES4A, MTOR, PARP8* mutations	Cisplatin, paclitaxel or carboplatin + paclitaxel	Response monitoring	[168]
Choudhuri et al., 2014	Stage IIIb/c	100	RT-PCR	Serum level	Carboplatin + paclitaxel	Response monitoring	[219]
Martignetti et al., 2014	Stage IIIc Serous Papillary	1	RT-PCR	FGFR2 fusion transcript	Carboplatin + paclitaxel (total 5 lines of treatment)	Response monitoring	[220]
Pereira et al., 2015	Stage I-IV Serous	22	WES, ddPCR, TGS	*TP53, PTEN, PIK3CA, MET, KRAS, FBXW7, BRAF* mutations	Platinum + taxane agent	Response monitoring	[163]
Piskorz et al., 2016	HGSOC	18	NGS	*TP53* mutation	Platinum agent	Response monitoring	[91]
Parkinson et al., 2016	Relapsed HGSOC	40	Digital PCR	*TP53* mutation	Heterogeneous standard of care treatment	Response monitoring	[164]
Flanagan et al., 2017	Relapsed Stage I-IV Serous	247	NGS	Methylation at CpG sites	Platinum agent	Response monitoring	[221]
Widschwendter et al., 2017	Stage I-IV HGSOC or Clear Cell	151	TUC-BS & RRBS	*COL23A1*, *C2CD4D* and *WNT6* candidate markers	Carboplatin + paclitaxel or carboplatin only	Response monitoring	[161]
Ratajska et al., 2017	Stage I-IV	121	NGS	*BRCA1/2* mutations	PARPi	Predict eligibility for PARPi	[222]
Christie et al., 2017	Stage I-IV HGSOC	30	NGS	*BRCA* reversion mutation	Platinum-based agent and/or PARPi	Predict treatment response	[213]
Weigelt et al., 2017	Stage III-IV	19	NGS	*BRCA* reversion mutation	Platinum-based agent ± taxane agent	Response monitoring	[214]
Giannopoulou et al., 2018	Stage I-IV HGSOC	50	RT-MSP	ESR1	Carboplatin + paclitaxel	Response monitoring	[153]
Du et al., 2018	Recurrent Stage II-III Serous	21	NGS	*TP53, BRCA1, NOTCH2, DNMT3A mutations and CNVs*	Platinum-based agent	Response monitoring	[165]
Morikawa et al., 2018	Stage I-IV Clear Cell	29	ddPCR	*KRAS, PIK3CA*	Carboplatin + paclitaxel ± docetaxel/carboplatin/gemcitabine/bevacizumab/CPT-11/irinotecan	Response monitoring	[223]
Arend et al., 2018	Stage III-IV HGSOC	14	NGS	50 gene Ion Torrent panel	Platinum + taxane agent	Response monitoring	[188]
Lin et al., 2019	High Grade Serous and Endometriod	97	NGS	*BRCA* reversion mutation, *TP53*	PARPi (rucaparib)	Predict primary and acquired resistance	[212]
Kim et al., 2018	Stage II-IV HGSOC	102	Sanger sequencing/Digital PCR	*TP53* mutation	Carboplatin + paclitaxel	Response monitoring	[189]
Oikkonen et al., 2019	HGSOC	12	NGS	*ERBB2* amplification	Platinum + taxane agent ± trastuzumab	Response monitoring	[224]
Iwahashi et al., 2019	Stage I-IV HGSOC, Mucinous, Endometrioid	4	CAPP-seq	*TP53, APC, BRCA1* and *KRAS* mutations	Carboplatin + paclitaxel	Response monitoring	[225]
Noguchi et al., 2020	Stage III-IV HGSOC	10	CAPP-seq	gene mutation profiles, blood tumor mutation burden	Carboplatin + paclitaxel ± bevacizumab	Response monitoring	[226]
Han et al., 2020	Stage III-IV EOC	10	NGS	88 genes panel (Axen Cancer Panel 1)	Standard chemotherapy	Response monitoring	[227]
Alves et al. 2020	Stage I-IV Serous, Clear Cell, Mucinous	11	qPCR	Level	Carboplatin + paclitaxel, gemcitabine, doxorubicin + bevacizumab or rucaparib (PARPi)	Response monitoring	[228]

Other somatic mutations seen more frequently in ovarian cancer may be used for monitoring response to treatment. As the main driver of clonal pathogenic lesions, *TP53* mutations are in nearly all primary ovarian tumour cells, synchronous tumours, and metachronous lesions. Detection of *TP53* mutations was therefore hypothesized to be a suitable biomarker for disease monitoring. Since the feasibility of sequencing cfDNA fragments to detect tumour-associated *TP53* mutation was first reported more than 15 years ago, studies have demonstrated that *TP53 *mutation detection can evaluate tumour burden, predict time to progression (TTP) and detect tumour recurrence [58, 91, 162–165, 189, 229, 230]. Similarly, another study that used whole-exome sequencing of plasma cfDNA to serially quantify allele fractions (AF) in plasma identified an increased representation of mutant alleles associated with the development of treatment resistance in three ovarian cancer patients. The researchers found that increased mutant AF of *RB1* and *ZEB2* was associated with cisplatin resistance, while *MTOR*, *CES4A* and *BUB1* mutations were associated with paclitaxel and carboplatin resistance, and *PARP8 *mutation was associated with liposomal doxorubicin resistance [168].

In terms of other mutations, Forshew et. al reported the emergence of a de novo EGFR tyrosine kinase mutation in an HGSOC patient at disease relapse that was not present 15 months earlier in the initial tumour sample [162]. This patient was identified to be eligible for treatment with gefitinib or erlotinib, however, the treatment efficacy and final clinical outcome were not reported. The study authors used tagged-amplicon deep sequencing (TAm-Seq) and screened 5995 genomic bases for low-frequency mutations to identify cancer mutations present in cfDNA at allele frequencies as low as 2%. More recently, Oikkonen et al. conducted a prospective cohort study with 78 cfDNA samples collected from 12 HGSOC patients collected before, during and after platinum-based chemotherapy, and detected clinically actionable mutations in 7 (58%) of patients. In one patient with ERBB2 amplification detected in ctDNA following the development of resistance to platinum chemotherapy, treatment was changed to trastuzumab with reduced dose carboplatin and dose-dense paclitaxel, with significant tumour shrinkage and complete normalization of CA-125 achieved [224]. Table 3 summarizes the studies investigating the clinical application of ctDNA for predicting and monitoring response to treatment in ovarian cancer.

## Conclusions and future directions

There is emerging evidence supporting the potential of liquid biopsy to enhance ovarian cancer management with the goal of improving survival. In the last decade, advances made in molecular analysis technologies have allowed for increased clinical applications of liquid biopsy in the diagnosis, prognosis, and prediction of response to treatment in ovarian cancer. The advantages over traditional biopsies include the ability for earlier detection of cancer and identification of microscopic minimal residual disease following primary debulking surgery, which are important considerations in ovarian cancer due to the possibility for earlier intervention and improved survival outcomes. Liquid biopsy can also capture the heterogeneity of ovarian tumours more comprehensively compared to conventional tissue biopsy. The non-invasiveness and feasibility of liquid biopsy allow for serial sampling and longitudinal monitoring to identify treatment resistance with tumour evolution over time and guide the selection of personalized therapy.

Currently, several challenges remain to be addressed prior to more routine utilization of liquid biopsy in ovarian cancer. For example, cfDNA analysis has limited sensitivity and specificity for early-stage ovarian cancer. Consequently, genetic mutations detected in the cfDNA or ctDNA should only be used to guide clinical decision-making in conjunction with other biomarkers and imaging techniques to improve the overall sensitivity and specificity. In addition, given the low proportion of ctDNA in cfDNA, optimization of current isolation strategies and improved sensitivity of analysis techniques are required to enhance the extraction yield and enable ctDNA detection at low allele frequencies. Another limitation is the current lack of reproducibility of liquid biopsy assays due to the lack of standardization across different laboratory workflows. Future studies should estimate the technical robustness and reproducibility of proposed biomarkers within and between laboratories by implementing a standardized procedure. Further studies examining the specific mechanisms of release, tissue of origin and biological significance are also required for most liquid biopsy components prior to clinical use.

Another challenge is to elucidate the multifactorial processes implicated in the development of chemoresistance that contributes to the high mortality rates in ovarian cancer. During treatment, regular monitoring with liquid biopsy can elucidate drug resistance acquired from genetic alterations, which may be present but undetectable with conventional approaches. The implications of oncogene expression on cancer drug resistance remain poorly understood, although there have been over 25 oncogenes including *KRAS, ERBB2, PIK3CA, AKT *hypothesized to contribute to drug resistance in ovarian cancer through various signalling pathways [215, 216, 224]. Genomic-based drug response prediction may offer a new horizon in ovarian cancer to improve clinical management. However, previous studies on drug resistance have been limited to small samples from certain ovarian cancer cell lines and have focused largely on ctDNA analysis [231–233]. In the future, more studies are required to examine the utility of liquid biopsy in predicting treatment response among different ovarian cancer subtypes and to compare the efficacy of the various liquid biopsy analytes. Further efforts to standardize analysis platforms and incorporate liquid biopsies as a companion biomarker in large-scale drug trials are also warranted.

In conclusion, liquid biopsy has emerged as a promising alternative to traditional tissue sampling methods for its potential utility in the earlier detection and management of ovarian cancer. However, current evidence suggests that liquid biopsy may be best used as a second-line or complementary diagnostic tool rather than the sole biomarker in deciding clinical management. To date, most studies evaluating liquid biopsy techniques are limited by small sample sizes and larger, higher-quality studies are required to provide more rigorous evidence prior to their routine clinical application.

## Data Availability

Not applicable.
